# Supplementary material for: Genome binning of viral entities from bulk metagenomics data
Source: Nat Commun. 2022 Feb 18;13:965. doi: 10.1038/s41467-022-28581-5 (PMC8857322; doi:10.1038/s41467-022-28581-5)
Supplement: Supplementary file 1 — Supplementary Information [file 41467_2022_28581_MOESM1_ESM.pdf]

Supplementary information

A combined deep learning pipeline for metagenomics binning

Authors

Joachim Johansen[1,2], Damian R. Plichta[2], Jakob Nybo Nissen[1,3], Marie Louise Jespersen[1,4], Shiraz A. Shah[5], Ling Deng[6], Jakob Stokholm[5,6], Hans Bisgaard[5], Dennis Sandris Nielsen[6], Søren J. Sørensen[7], Simon Rasmussen[1].

Affiliations

[1] Novo Nordisk Foundation Center for Protein Research, Faculty of Health and Medical Sciences, University of Copenhagen, Copenhagen, Denmark

[2] Infectious Disease and Microbiome Program, Broad Institute of MIT and Harvard, Cambridge, MA, USA.

[3] Statens Serum Institut, Viral & Microbial Special diagnostics, Copenhagen, Denmark

[4] National Food Institute, Technical University of Denmark, Kongens Lyngby, Denmark

[5] Copenhagen Prospective Studies on Asthma in Childhood (COPSAC), Herlev and Gentofte Hospital, University of Copenhagen, Copenhagen, Denmark

[6] Section of Food Microbiology and Fermentation, Department of Food Science, Faculty of Science, University of Copenhagen, Copenhagen, Denmark

[7] Section of Microbiology, Department of Biology, University of Copenhagen, Copenhagen, Denmark

Author List Footnotes

Correspondence: Simon Rasmussen (simon.rasmussen@cpr.ku.dk)

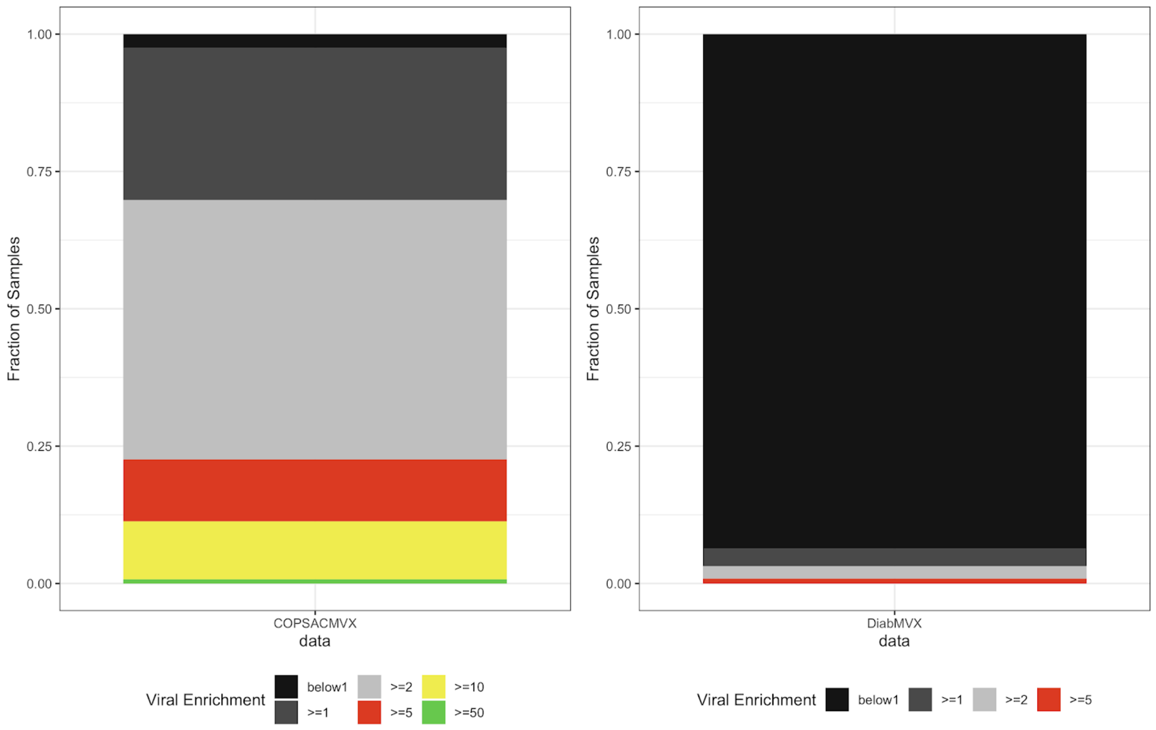

Supplementary Figure 1. Virome QC evaluation of metaviromes. All samples in COPSAC and the Diabimmune T1D metavirome datasets were processed with ViromeQC to achieve viral enrichment estimates. The magnitude of enrichment relative to bulk metagenomics is coloured using breaks described in the paper by Zolfo *et al.* where black indicates low and green indicates high enrichment.

Virsorter2 on Diabimmune T1D

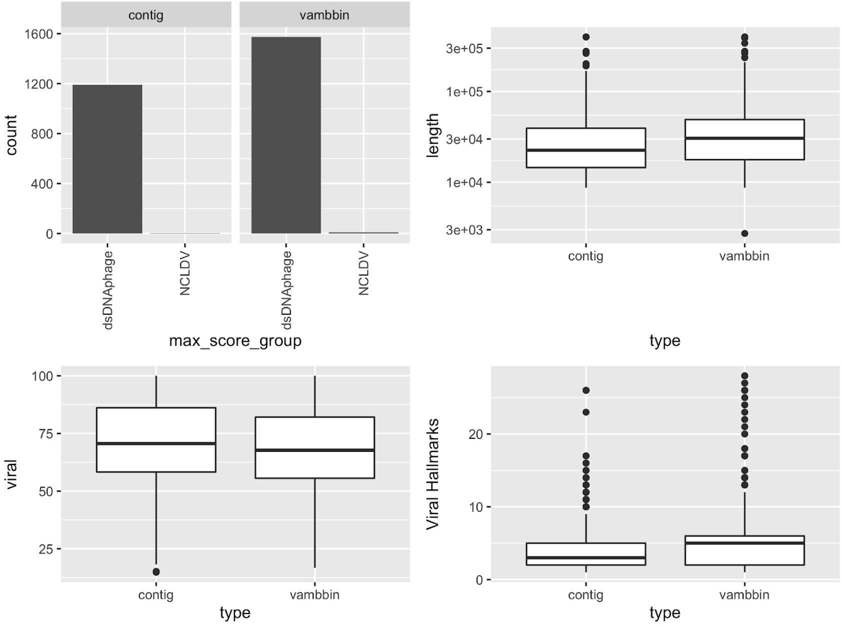

Virsorter2 on COPSAC dataset

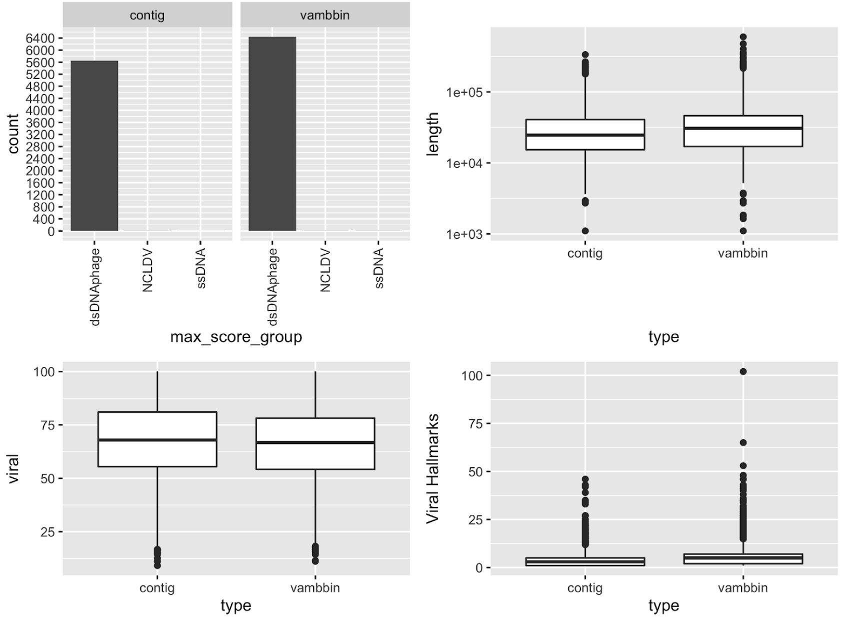

Supplementary Figure 2. Virsorter2 prediction statistics. Virsorter2 was run on sequences assembled and binned from COPSAC and Diabimmune bulk metagenomes. Prior to Virsorter2 analysis, sequences were cleaned using CheckV that removes bacterial regions. Results are only shown for sequences predicted as double or single-stranded DNA phage or nucleocytoplasmic large DNA viruses (NCLDV) with a prediction score >0.9 and >=1 viral hallmark gene. In COPSAC this corresponded to 6459 vamb bins and 5654 contigs. And in Diabimmune we analysed 1581 vamb bins and 1194 contigs.

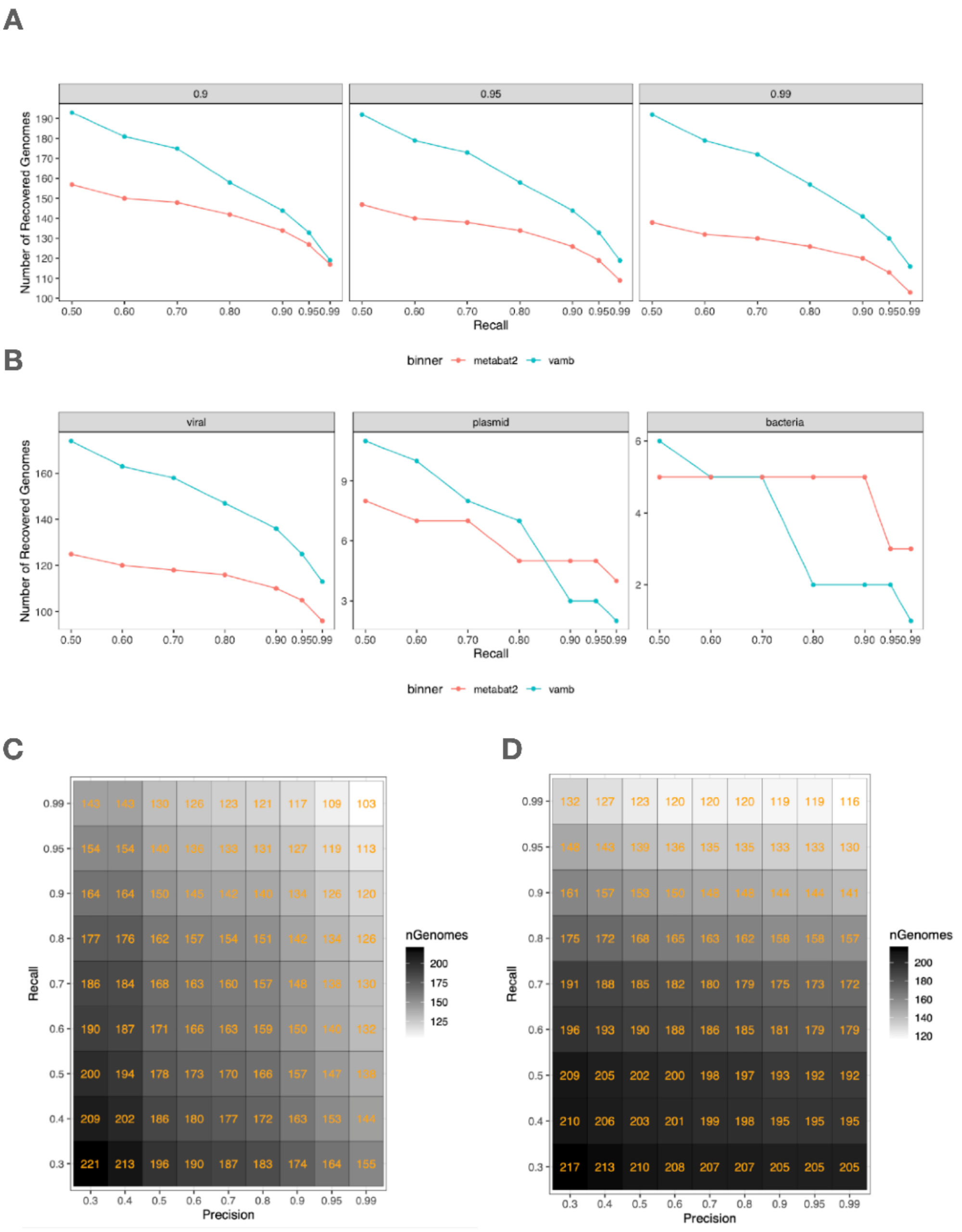

Supplementary Figure 3. Evaluating VAMB and MetaBAT2 for binning phages on simulated data. A) The number of genomes binned by VAMB and MetaBAT2 with increasing Recall and Precision at three levels 0.9, 0.95, 0.99, separated by organisms viral, plasmid and bacteria. B) The number of genomes binned by VAMB and MetaBAT2 with increasing Recall and Precision > 0.95 for viruses, plasmids and bacteria in their own panel. Heatmap showing the number of genomes binned by VAMB (C) and MetaBAT2 (D) in the Recall and Precision range [0.3-0.99].

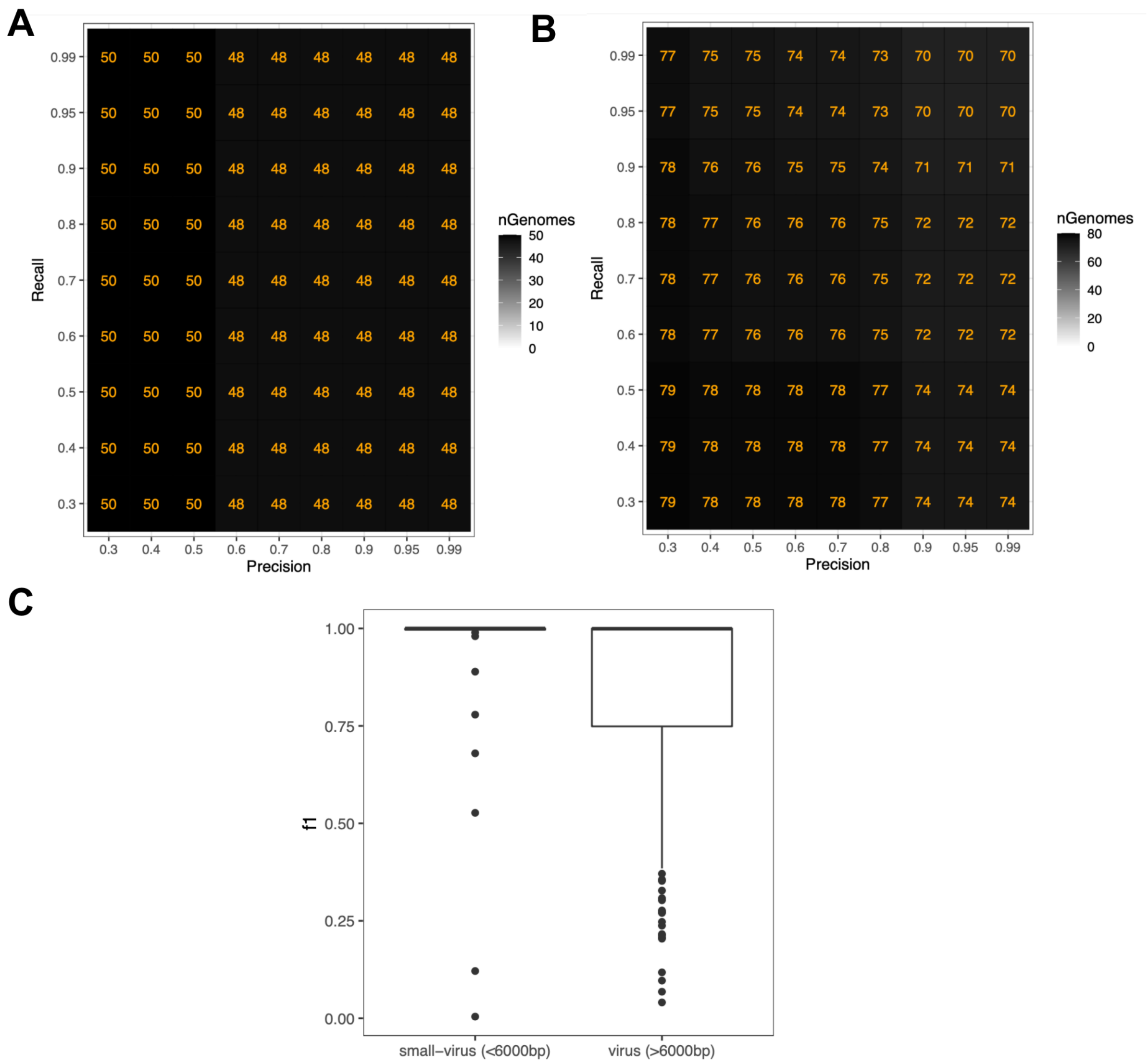

Supplementary Figure 4. Performance of VAMB binning the phage only simulated datasets and small-viruses. A) Precision and recall performance of VAMB binning on small phage genomes (<6,000 bp, n=50). B) Precision and recall performance of VAMB binning on crass-like (n=80) viruses. C) F1-score distributions of small virus genomes and larger virus genomes (>6000 bp, n=230) in the mixed genome dataset.

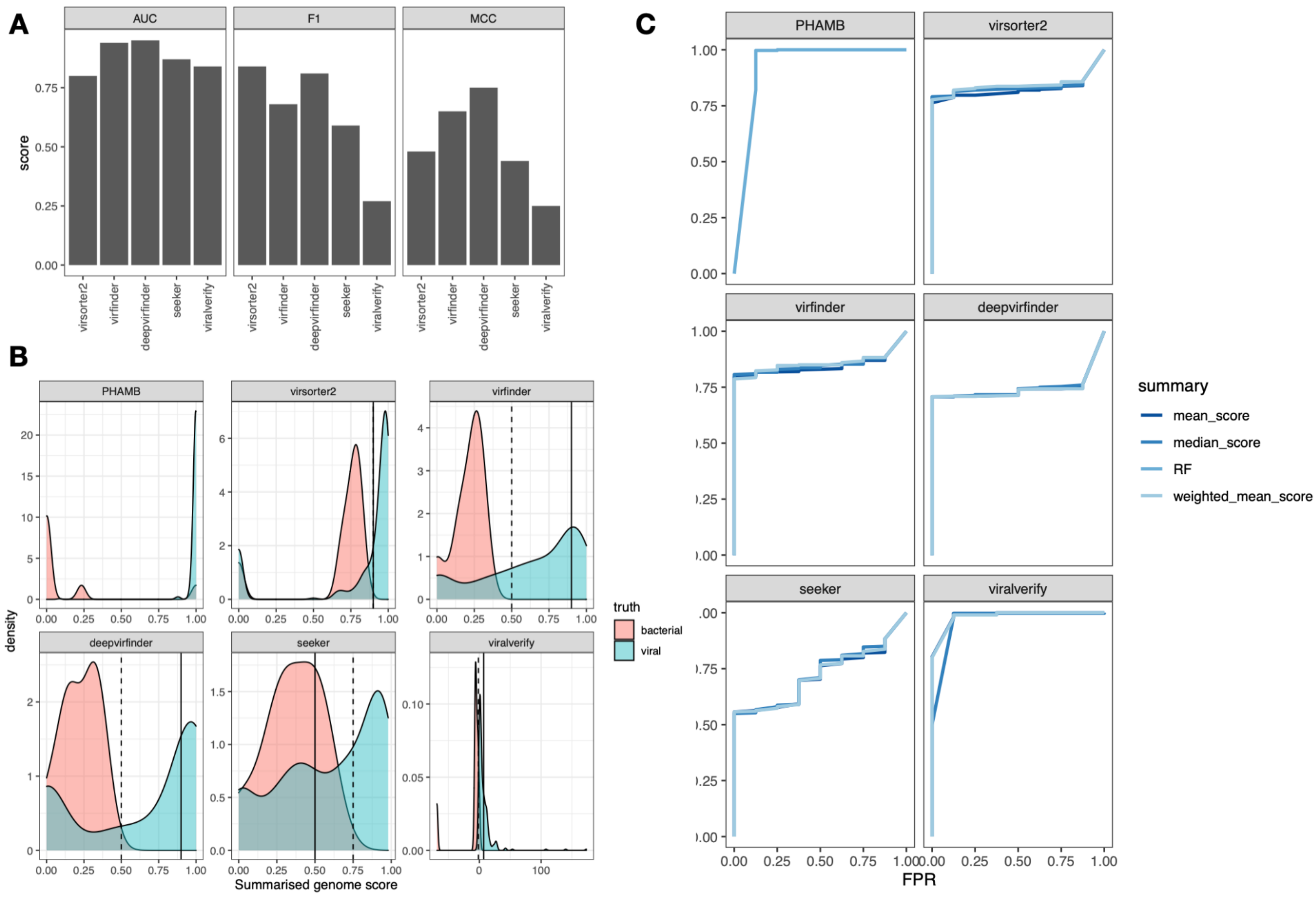

Supplementary Figure 5. Evaluation of viral predictors on simulated data. A) Viral prediction performance on contigs by published viral contig predictors. In (A) AUC, F1 and MCC were calculated on viral or non-viral fragmented contigs from CAMI simulated dataset with genomes from viruses and bacteria (see methods for cutoffs). In (B) Density plots of summarised genome scores for simulated CAMI genomes. The summarised genome scores are displayed for each virus prediction tool (length weighted mean score) and PHAMB (probability score) then colored by the ground truth genome label. C) ROC performance-curves shown for each method. All results here were calculated based on the simulated mixed genome dataset.

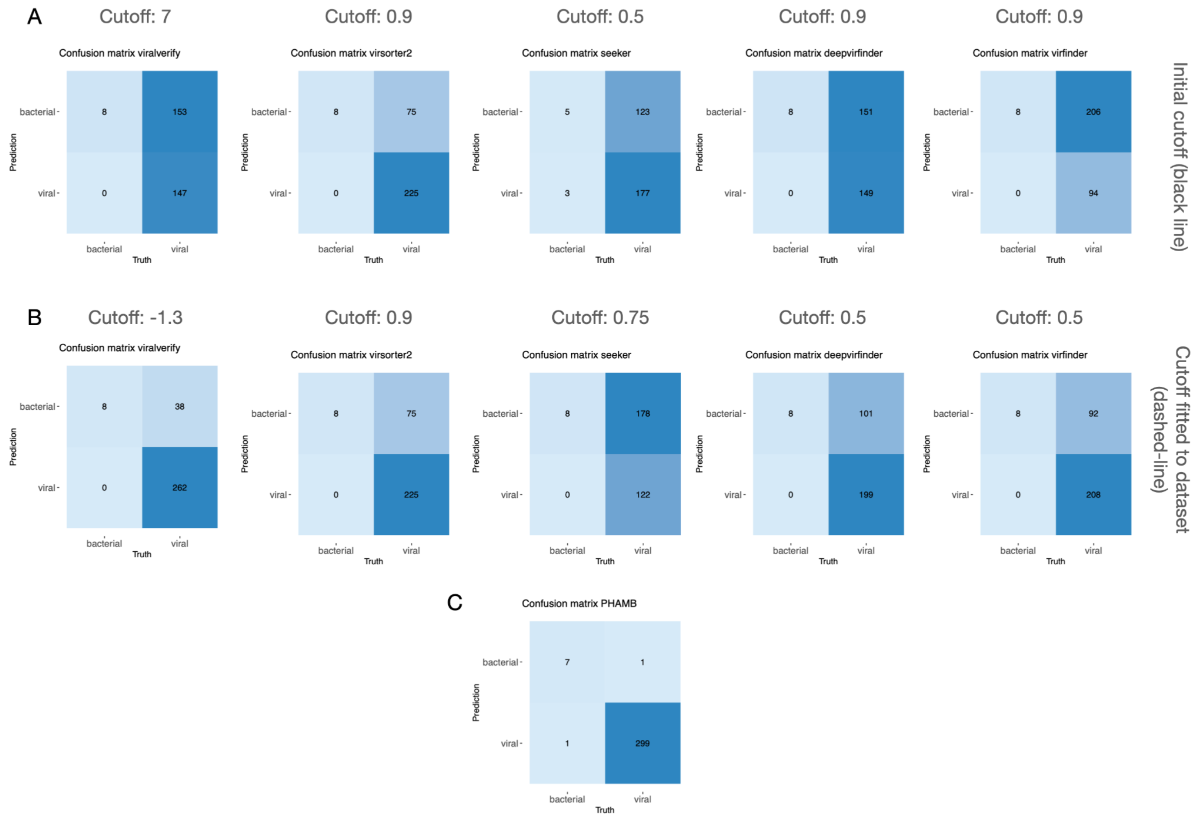

Supplementary Figure 6. Confusion matrices for viral predictors on simulated data with initial cutoffs and fitted cutoffs. Confusion matrices of predictions for simulated CAMI genomes. First row (A) shows the confusion matrices of each virus prediction tool based on an initial cutoff and second row (B) shows the confusion matrices based on ideal cutoffs on the data. In (C) the confusion matrix for PHAMB is displayed

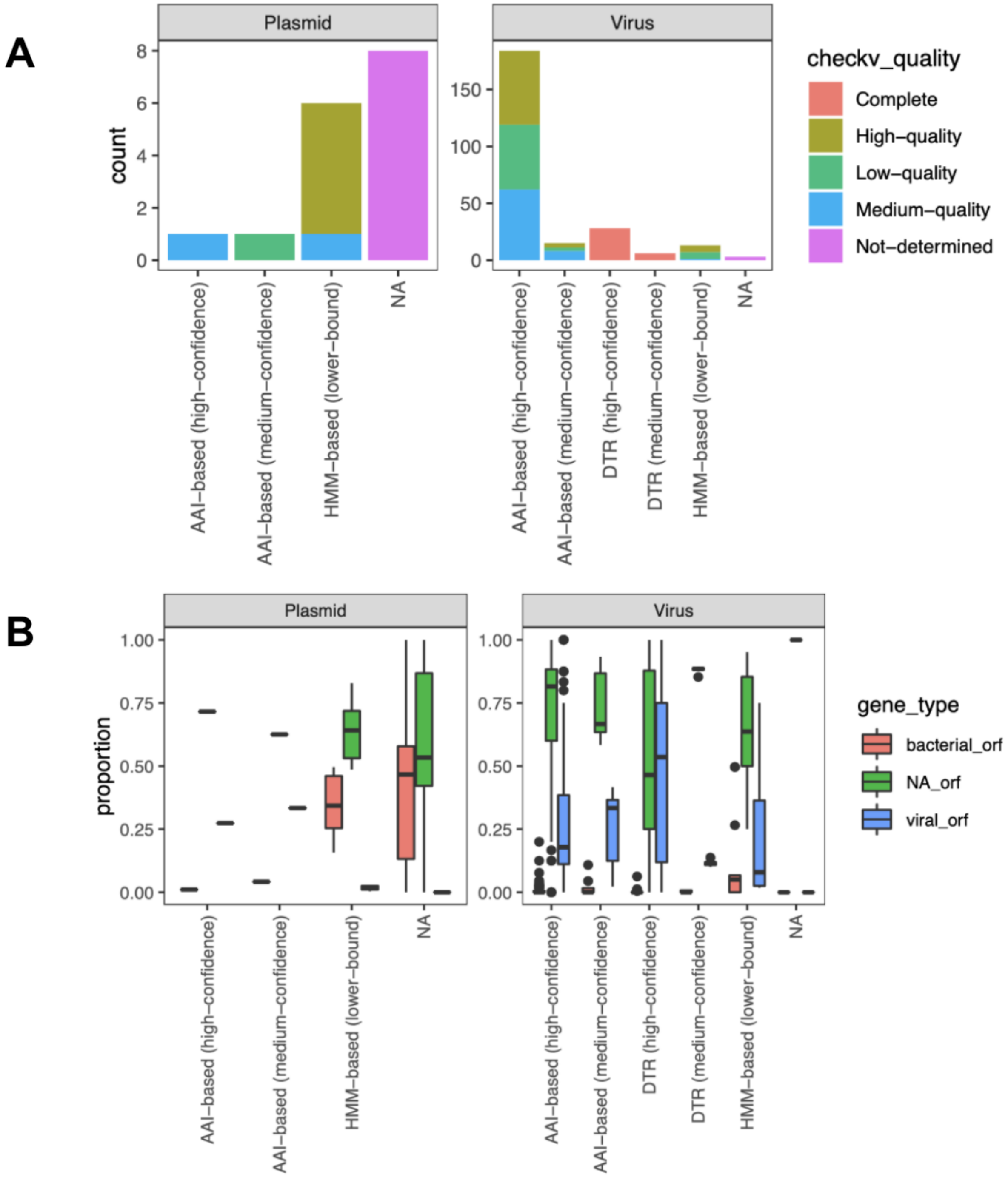

Supplementary Figure 7. CheckV evaluation of binned virus and plasmids from the mixed simulated dataset. A) CheckV quality evaluation counts of plasmid (n=16) and virus genomes (n=249). B) Boxplot of the proportions of viral (viral_orf), host (bacterial_orf) and unknown genes (NA_orf) in plasmids and virus genomes. Each distribution is separated based on the CheckV quality evaluation assigned to each bin. Results of (A) and (B) were calculated based on the simulated mixed genome dataset.

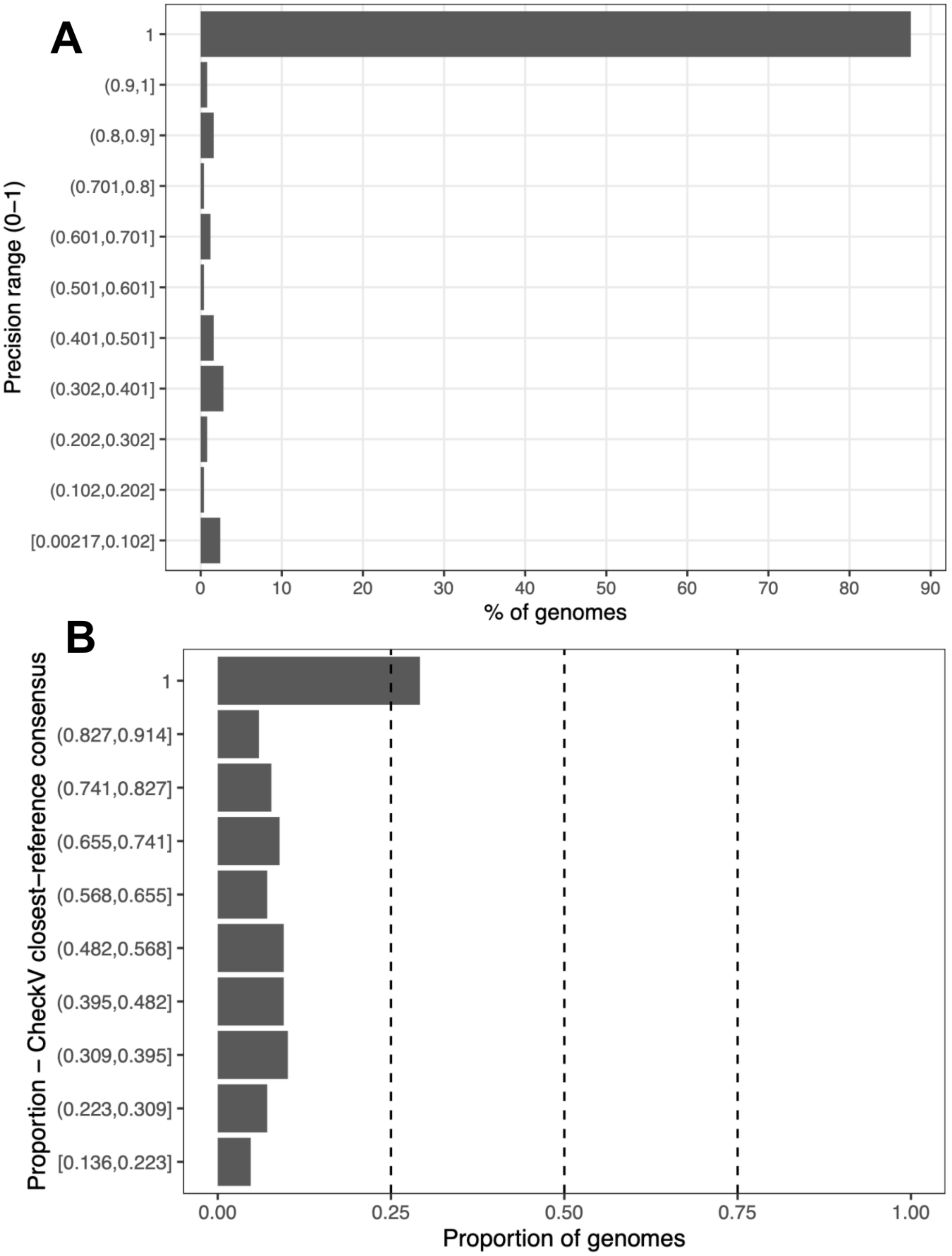

Supplementary Figure 8. Contamination in simulated viral-bins and nearest reference consensus. A) Viral bin precision/degree-of-contamination for binning using VAMB on the simulated mixed genome dataset (bacteria n=8, plasmids n=20 and viruses n=280). Here the vast majority of bins has a Precision of 1 meaning that all contigs originate from the same genome B) The maximum nearest-reference contig consensus (in the CheckV database) within viral bins of the simulated mixed genome dataset. A proportion of 1 indicates that all contigs in a bin match the same closest-reference genome.

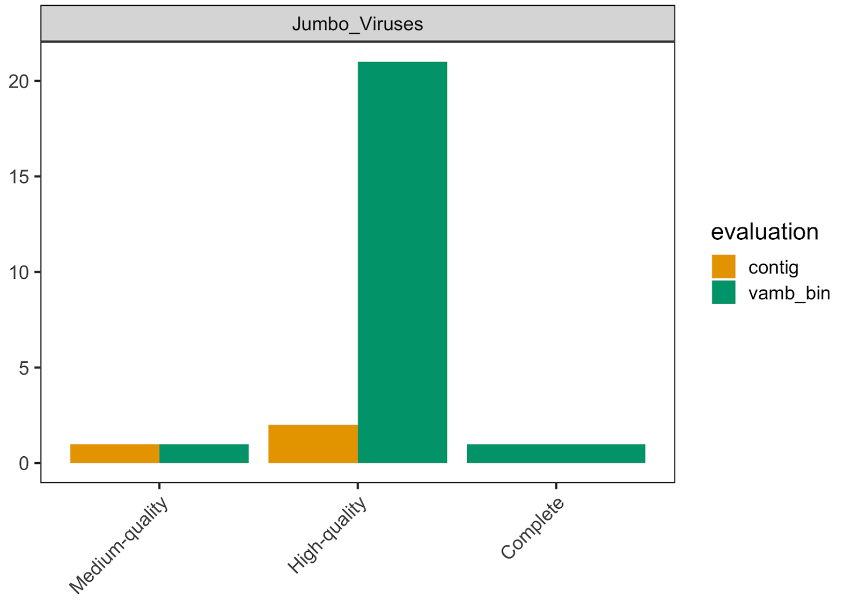

Supplementary Figure 9. Jumbo viruses in HMP2. Viral completeness was estimated for both single-contigs and VAMB-bins in HMP2. Evaluation of genome completeness was determined using CheckV here shown for Medium-quality >=50% , High-quality >=90%, Complete =100%). Closed genomes are annotated as “Complete” based on direct terminal repeats or inverted terminal repeats. After adjusting for possible host-contaminating sequences in both single-contigs and VAMB bins, several Jumbo viruses with a size >=200 kbp were found.

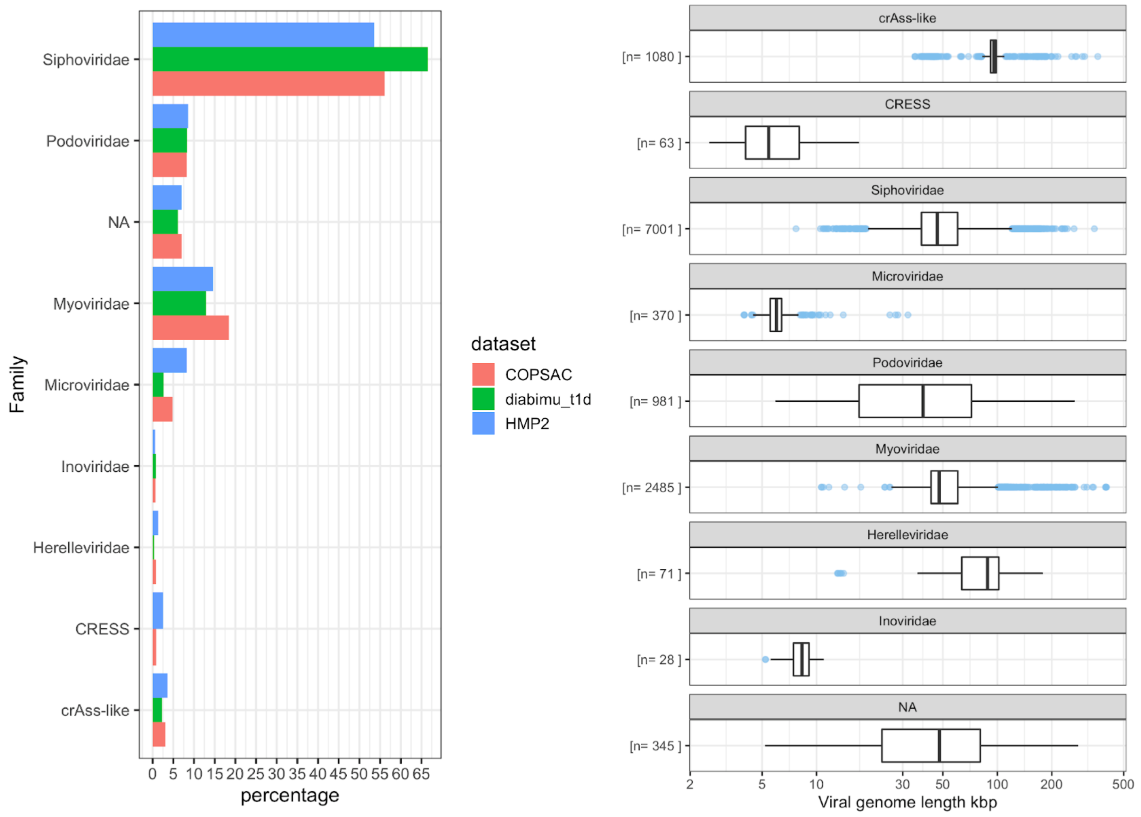

Supplementary Figure 10. Viral taxonomy percentages for datasets. Viral taxonomy was assigned to each bin using the plurality rule described before in Roux et al.: (1) taxonomy was assigned to bins with at least two PVOG proteins (VOGdb) using a majority vote (>=50% else NA) on each taxonomic rank based on the last common ancestor (LCA) annotation from the PVOG entries. (2) The CheckV VOGclade taxonomy was transferred if available from the best viral genome match in the CheckV database. CrAss-like viruses were annotated as described by Guerin *et al.* By far, the most frequently annotated viral family was *Siphoviridae* (n=7001) in all datasets, the remaining bins were annotated as following crAss-like (n=1080), CRESS (n=63), *Microviridae* (n=370), *Podoviridae* (n=981), *Myoviridae* (n=2485), *Herelleviridae* (n=71), *Inoviridae* (n=28).

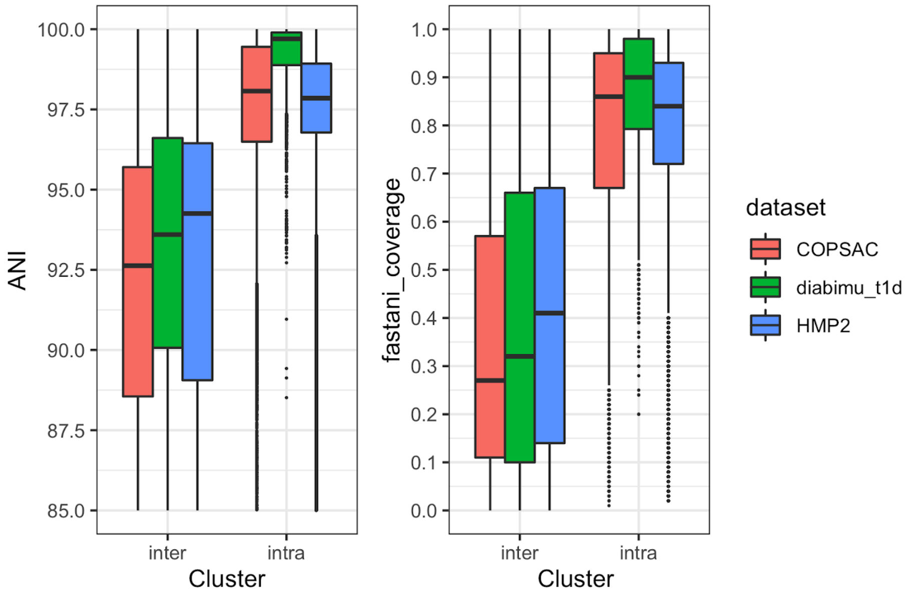

Supplementary Figure 11. Average nucleotide identity (ANI) distributions. In order to assess how VAMB clusters highly similar viral bins, we compared ANI between viral bins of different VAMB clusters (inter) and within the same cluster (intra) in COPSAC (n=5215 vambbins), Diabimmune (n=1066 vambbins) and HMP2 (n=17344 vambbins) dataset. Coverage was also calculated in a similar way between bins as the number of bidirectional fragments / total fragments. This showed that highly similar viral bins (ANI > 97.5) were consistently clustered into the same VAMB clusters.

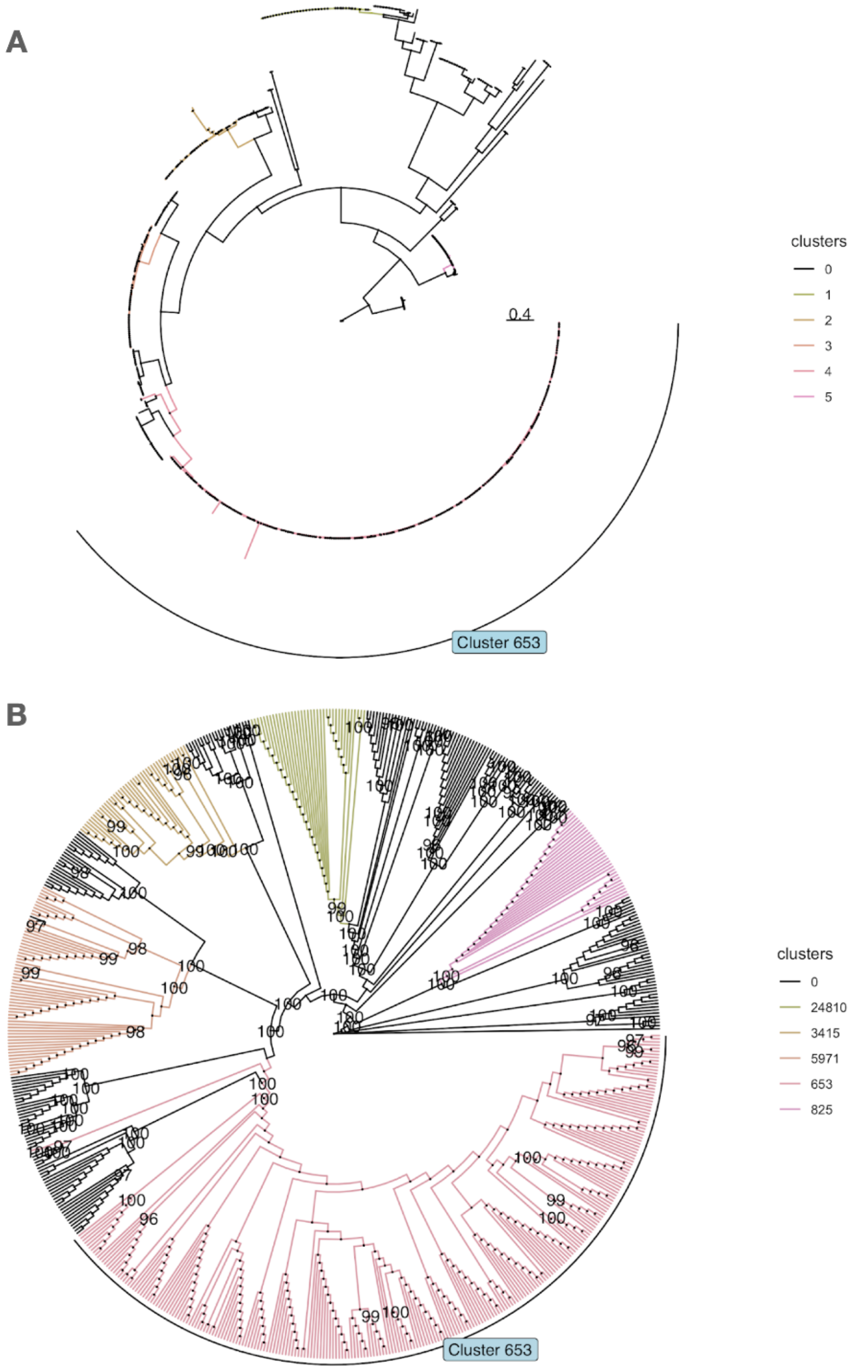

Supplementary Figure 12. Phylogenetic tree of crAss-like viruses. (a) A phylogenetic tree was constructed for crAss-like viral bins identified in the HMP2 dataset based on proteins annotated as the large terminase subunit protein (the *terL* gene). Branches drawn according to phylogenetic distance with cluster 653 indicate the progenitor-crassphage. **(b)** The cladogram of the tree, same as Figure 3d, but displayed with bootstrap values calculated by IQtree.

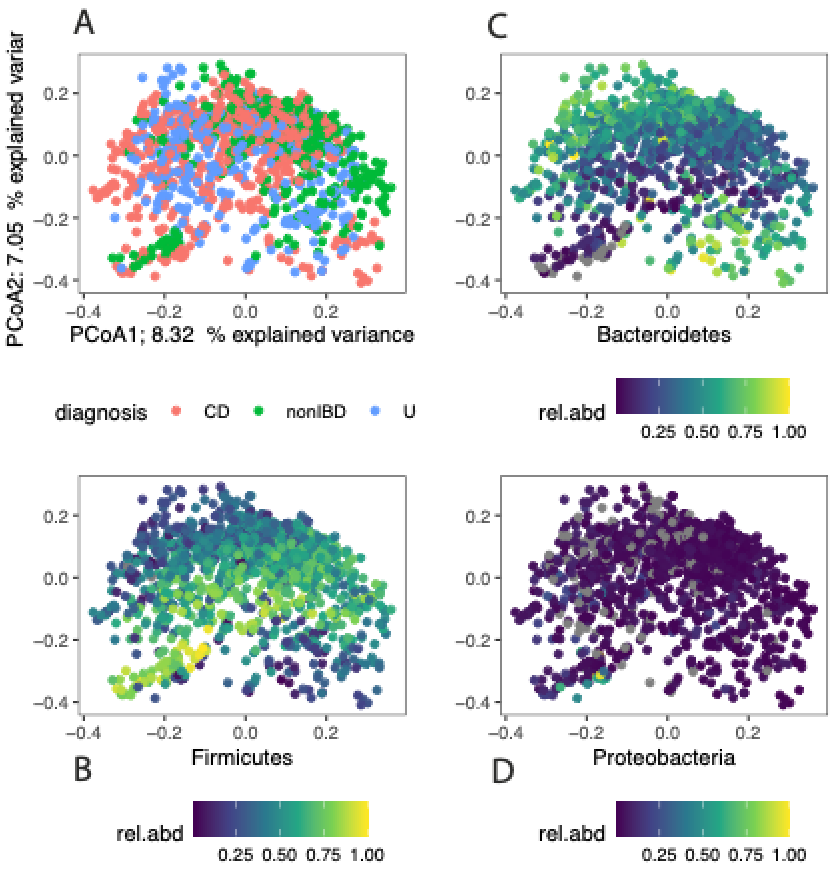

Supplementary Figure 13. Principal component analysis (PCoA) based on MAGs in HMP2. (a) PCoA was performed on Bray-Curtis distance matrix of MAG abundances in the HMP2 cohort. Samples were not easily separated based on diagnosis status from the HMP2 metadata **(b-d)** Instead, differences in the proportion of relative abundance occupied by Bacteroidetes and Firmicutes bacteria was reflected by the PCoA analysis.

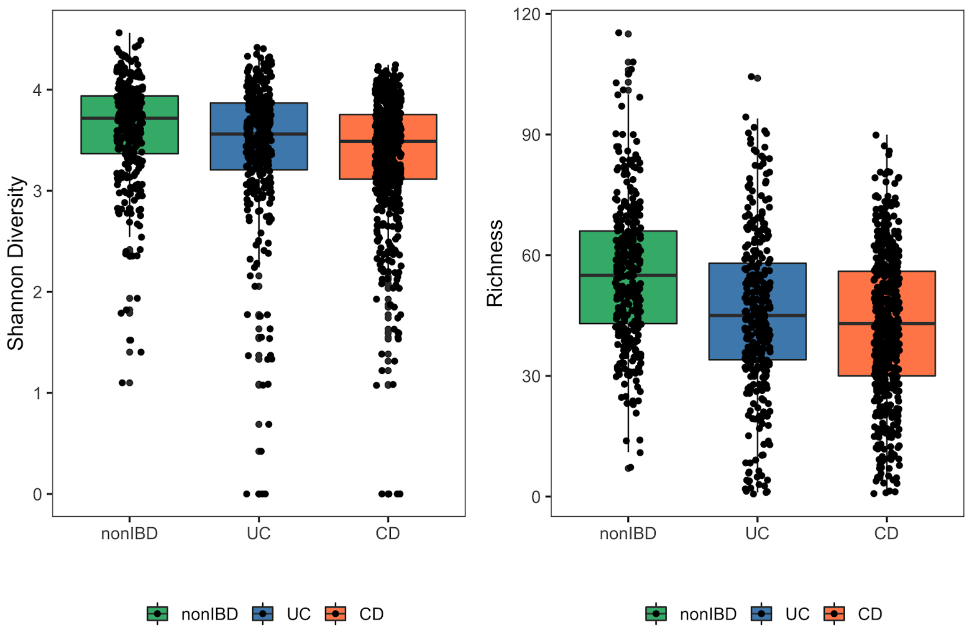

Supplementary Figure 14. Bacterial alpha diversity metrics of the HMP2. The bacterial composition of HMP2 including nonIBD (n=326), UC (n=UC) and CD (n=573) samples were characterised with alpha-diversity metrics such as Shannon Diversity index (vegan package in R) and Richness (the number of different MAGs with abundance above zero). A general downward shift in both metrics was observed for samples from subjects with diagnosed ulcerative colitis (UC) and Crohn's disease (CD) relative to samples from nonIBD (control) subjects.

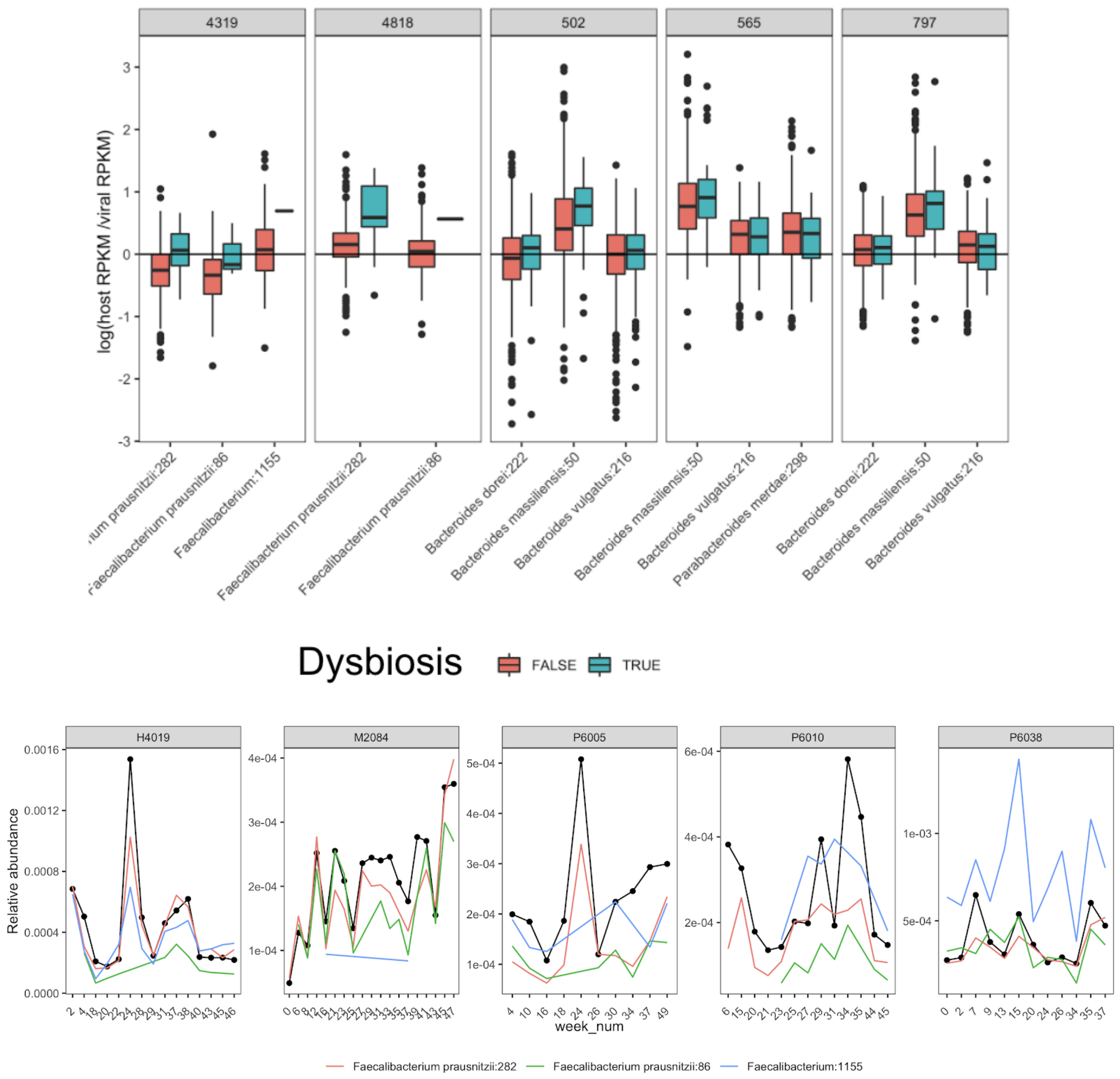

Supplementary Figure 15. Abundance patterns of associated Viruses and MAGs. (Upper panels) Viral-bacterial relationships were determined using a combination of CRISPR-spacer and sequence alignment. In order to confirm host-dependencies for putative temperate viruses (each panel), the ratio of relative abundance was calculated across samples for a given virus and bacterial host(s). Furthermore, these abundance ratios were illustrated for dysbiotic samples (n=210) vs non-dysbiotic samples (n=995) to capture potential shifts or breaks in host-dependency. **(Lower panels)** The relative abundance of individual viruses (viral *cluster 4319* indicated with the black line) can be visualised along associated bacterial hosts (coloured lines) over time in different subjects (each panel).

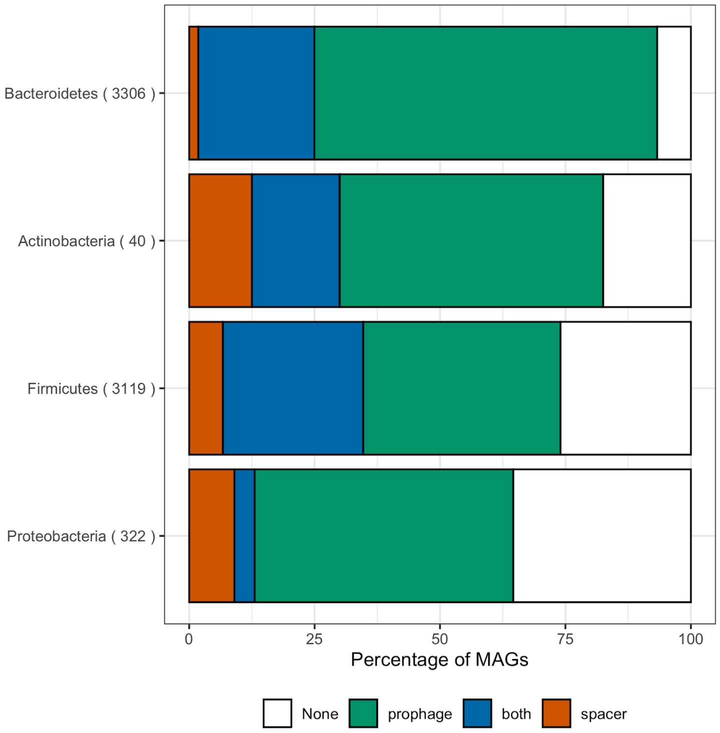

Supplementary Figure 16. MAGs connected to Viruses by Phylum in HMP2. Host-viral associations were determined using CRISPR spacers, which were mined from MAG bins, and viral bin alignments to MAG bins. The percentage of bacterial bins annotated with a viral population is illustrated below on phylum rank. Here the majority of Bacteroidetes bins are only annotated to a virus through viral alignment as *prophage* evidence (green), second is where *both* (blue) a viral alignment and CRISPR spacer match the given virus. Finally, some bacterial bins only have CRISPR *spacers* (red) against viruses and no viral alignment.

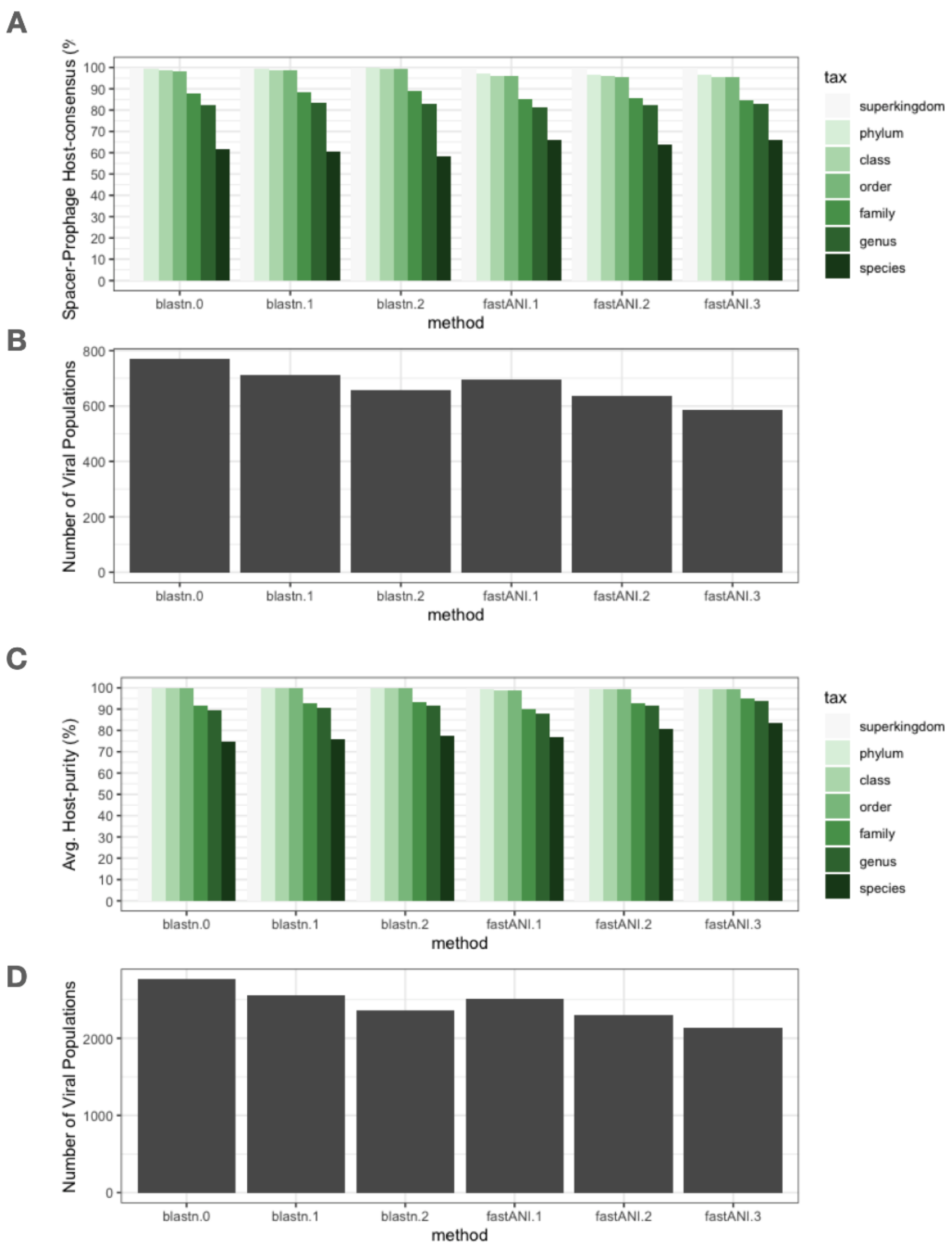

Supplementary Figure 17. Viral-host prediction benchmark on the HMP2 dataset.

(a) For viruses with hosts predicted by both methods (genome-alignment and CRISPR-spacers), the average host-prediction consensus was calculated across multiple taxonomic ranks. The consensus was calculated using three different cutoffs with blastn and one cutoff for FastANI. In addition, the number of viral populations host annotated at each threshold is shown in **(b)**. We also calculated a "purity" measurement of predicted hosts for each virus in **(c)**. I.e. if a virus genome aligns to three different MAGs and MAG taxonomy on species rank is [B. vulgatus, B. vulgatus, B. dorei] the virus host purity is 66% on species rank. For this benchmark the number of viral populations used is displayed in **(d).**

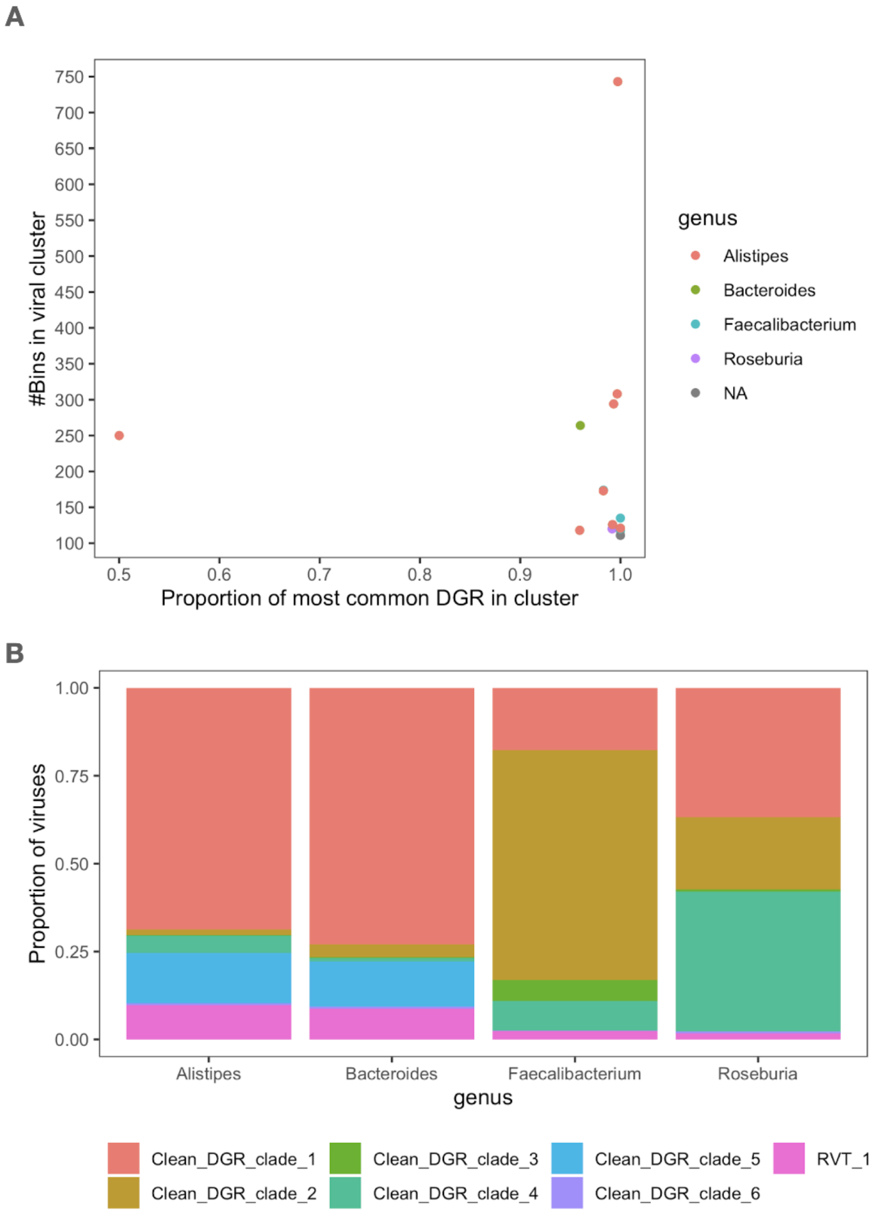

Supplementary Figure 18. Diversity generating region (DGR) specificity. (a) DGR specificity, the most common DGR type within a VAMB cluster, for viral populations coloured by predicted host taxonomy on genus rank. The DGR-clade for reverse transcriptase (RT) proteins were characterised using methods described in Roux et al., 2020. The most frequent DGR was calculated for each viral population to determine DGR specificity. (**b)** DGR-specificity by viral host taxonomy on genus rank.

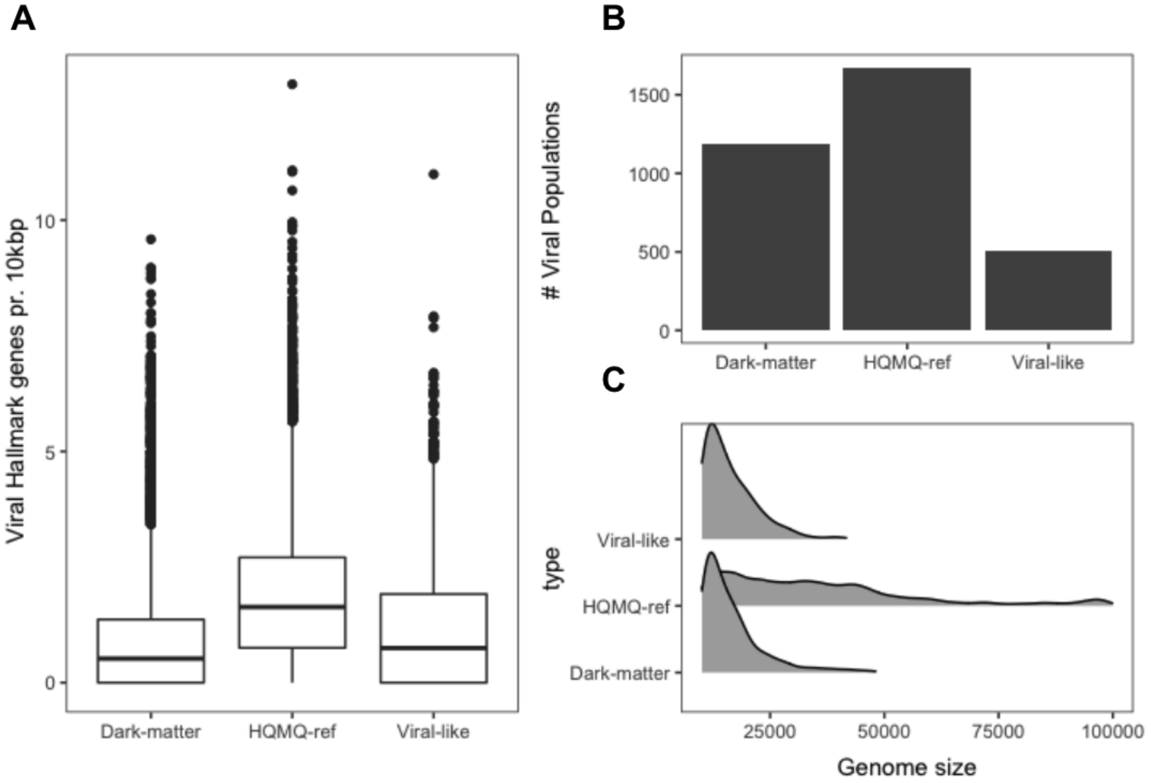

Supplementary Figure 19. Virsorter 2 predictions for Viruses and Dark-matter groups in HMP2. VAMB clusters/populations were defined as HQMQ-ref (n=45.983) with a high-quality or medium-quality viral bin, else as Dark-matter (n=392.226). Dark-matter populations with CRISPR-spacers against a bacterial MAG were annotated as Viral-like (n=43.695). All bins within each group with a minimum size of 10 kbp were analysed using Virsorter revealing high prediction prediction scores for bins found in all groups (A). **(b-c)** The number of viral hallmarks was higher in the HQMQ group which was also the group with the highest number of bins with a score >0.75 (C). **(d)** In addition, the HQMQ group also comprises larger viruses, suggesting that Dark-matter and Viral-like contains smaller or fragmented viruses.

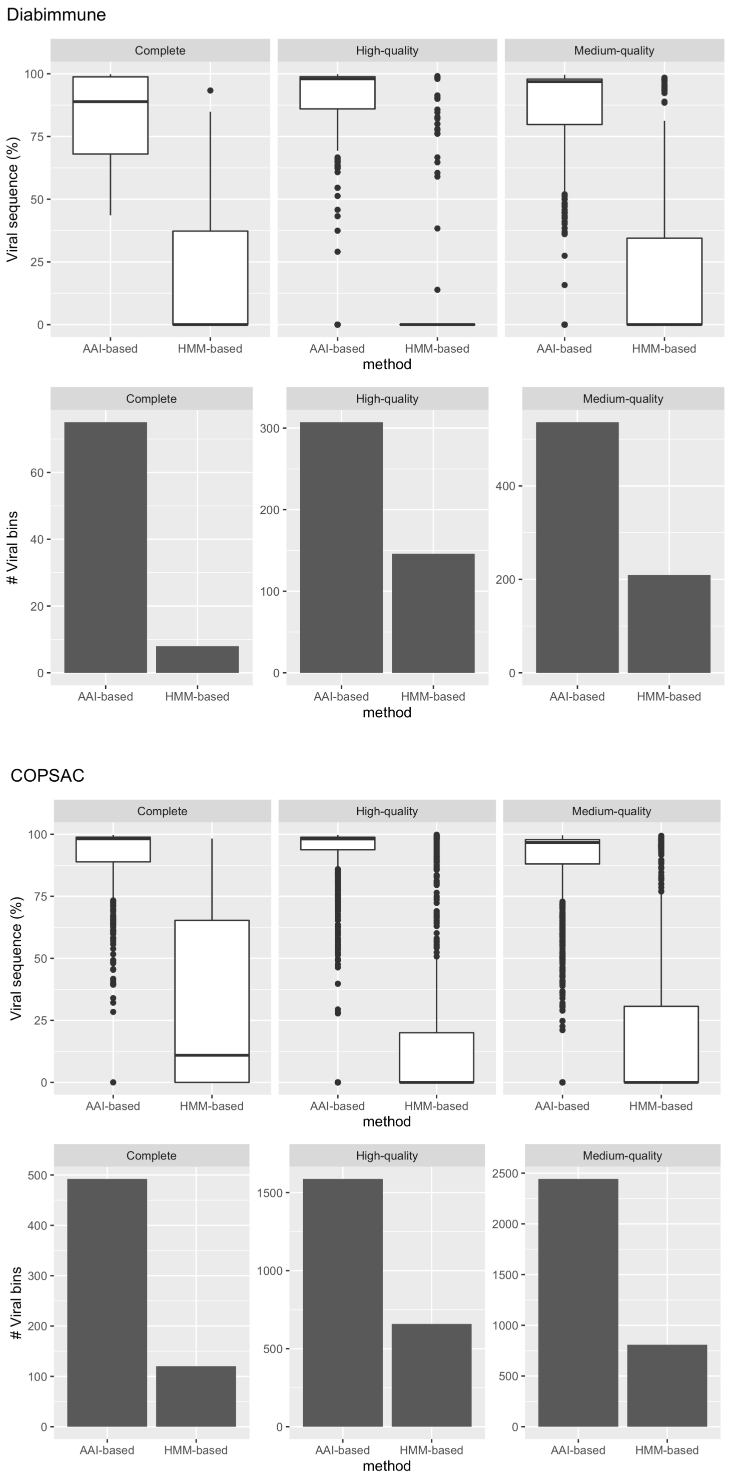

Supplementary Figure 20. Viral sequence proportions of VAMB bins in Diabimmune and COPSAC. The boxplot distributions for VAMB bins predicted by the AAI-based and HMM-marker model show the proportion of sequence in VAMB bins annotated as viral based on the presence of viral genes according to CheckV. These boxplots are organised according to the predicted CheckV quality tier and the number of bins in each distribution is shown as a barplot below. In COPSAC: Complete (492 AAI, 120 HMM), High-quality (1587 AAI, 658 HMM) and Medium-quality (2442 AAI, 807 HMM). In Diabimmune: Complete (75 AAI, 8 HMM), High-quality (307 AAI, 146 HMM) and Medium-quality (536 AAI, 209 HMM)

Supplementary Tables

Method	Summary Method	AUC	F1	MCC
**PHAMB**	**Random Forest**	**0.99**	**0.99**	**0.91**
**Viralverify**	**Weighted mean score**	**0.86**	**0.23**	**0.11**
**Virfinder**	**Weighted mean score**	**0.69**	**0.06**	**0.05**
**DeepVirFinder**	**Weighted mean score**	**0.6**	**0.23**	**0.11**
**Seeker**	**Weighted mean score**	**0.55**	**0.69**	**0.16**
**Virsorter2**	**Weighted mean score**	**0.3**	**0.35**	**0.13**

Supplementary Table 1. Random Forest (RF) performance table. The RF model was built and trained as described in Methods. AUC, F1-score and Matthews correlation (MCC) were calculated for prediction results on viral bins from Diabimmune. These performance scores were calculated based on probability scores from the trained RF model and summarised viral-bin scores of various viral prediction tools. For all tools except the RF model, genomes were labelled viral if the summarised viral-score across all contigs, calculated either as a mean, median or contig length weighted mean passed a threshold (in this table for the weighted mean score). The following thresholds used were 7, 0.5, 0.9, 0.9, 0.9 for Viralverify, Seeker, Virsorter2, Virfinder and DeepVirfinder, respectively.

Dataset	Evaluation	Overcompleteness	Count	Percentage
Diabimmune	contig	High-quality	151	96.17
Diabimmune	contig	Unsure (completeness > 120%)	6	3.82
Diabimmune	vamb_bin	High-quality	326	85.79
Diabimmune	vamb_bin	Unsure (completeness > 120%)	54	14.21
COPSAC	contig	High-quality	1080	95.83
COPSAC	contig	Unsure (completeness > 120%)	47	4.17
COPSAC	vamb_bin	High-quality	1812	87.88
COPSAC	vamb_bin	Unsure (completeness > 120%)	250	12.12
HMP2	contig	High-quality	2272	93.92
HMP2	contig	Unsure (completeness > 120%)	147	6.08
HMP2	vamb_bin	High-quality	6127	92.08
HMP2	vamb_bin	Unsure (completeness > 120%)	527	7.92

Supplementary Table 2. Overcomplete genomes VAMB bins vs single-contig evaluation. We investigated the frequency of viral bins with a viral size quite greater than the anticipated reference virus determined by the CheckV AAI-model. We tallied the number of High-quality bins determined and counted the ones with completeness > 120% for all datasets. This was also done for HQ single-contigs evaluated using the CheckV AAI-model.

Dataset	Family	# Genomes	# Populations	% of populations
COPSAC	crAss-like	143	38	3.11
COPSAC	CRESS	12	10	0.81
COPSAC	Herelleviridae	18	9	0.73
COPSAC	Inoviridae	15	8	0.65
COPSAC	Microviridae	71	59	4.83
COPSAC	Myoviridae	496	225	18.42
COPSAC	NA	99	86	7.04
COPSAC	Podoviridae	278	101	8.27
COPSAC	Siphoviridae	1536	685	56.10
Diabmmune	crAss-like	21	6	2.28
Diabmmune	Herelleviridae	1	1	0.38
Diabmmune	Inoviridae	2	2	0.76
Diabmmune	Microviridae	10	7	2.66
Diabmmune	Myoviridae	74	34	12.92
Diabmmune	NA	25	16	6.08
Diabmmune	Podoviridae	38	22	8.34
Diabmmune	Siphoviridae	356	175	66.54
HMP2	crAss-like	916	50	3.61
HMP2	CRESS	51	35	2.52
HMP2	Herelleviridae	52	19	1.37
HMP2	Inoviridae	11	8	0.58
HMP2	Microviridae	289	115	8.30
HMP2	Myoviridae	1915	202	14.57
HMP2	NA	221	96	6.93
HMP2	Podoviridae	665	119	8.59
HMP2	Siphoviridae	5109	742	53.53

Supplementary Table 3. Viral taxonomy counts for datasets. Viral bins were taxonomically annotated as described in the Methods section *Viral taxonomy and function*. In the table, the percentage of bins annotated to a given viral family is shown. In addition, the number of distinct viral populations annotated is also shown.

Supplementary Data 1 (excel file). Jumbo virus-bins CheckV table

Supplementary Data 2 (excel file). Counts of viral proteins

Supplementary Data 3 (excel file). Enriched viral proteins by predicted host taxonomy

Supplementary Data 4 (excel file). Metadata file for Genomes included in CAMISIM simulation.
