# Supplementary material for: Genome binning of viral entities from bulk metagenomics data
Source: Nat Commun. 2022 Feb 18;13:965. doi: 10.1038/s41467-022-28581-5 (PMC8857322; doi:10.1038/s41467-022-28581-5)
Supplement: Supplementary file 3 — Description of Additional Supplementary Files [file 41467_2022_28581_MOESM3_ESM.pdf]

Description of Additional Supplementary Files

File Name: Supplementary Data 1

Description: Putative Jumbo viruses.

File Name: Supplementary Data 2

Description: Viral proteins by host-genera.

File Name: Supplementary Data 3

Description: Enriched viral proteins by host-genera.

File Name: Supplementary Data 4

Description: Metadata file for Genomes included in CAMISIM simulation.
